# The Radiation-Specific Components Generated in the Second Step of Sequential Reactions Have a Mountain-Shaped Function

**DOI:** 10.3390/toxics11040301

**Published:** 2023-03-25

**Authors:** Katsuhito Kino

**Affiliations:** Faculty of Science and Engineering, Tokushima Bunri University, 1314-1 Shido, Sanuki-shi 769-2193, Kagawa, Japan; kkino@kph.bunri-u.ac.jp; Tel.: +81-87-899-7290

**Keywords:** radiation hormesis, LNT, radiation adaptive response, cancer incidence probability, Bateman equation, mountain shape, mathematic proof, sequential reaction

## Abstract

A mathematical model for radiation hormesis below 100 mSv has previously been reported, but the origins of the formula used in the previous report were not provided. In the present paper, we first considered a sequential reaction model with identical rate constants. We showed that the function of components produced in the second step of this model agreed well with the previously reported function. Furthermore, in a general sequential reaction model with different rate constants, it was mathematically proved that the function representing the component produced in the second step is always mountain-shaped: the graph has a peak with one inflection point on either side, and such a component may induce radiation hormesis.

## 1. Introduction

Cancer is a disease that afflicts people worldwide, and there is no specific cure for it. Cancer is generated by various causes, including radiation exposure. When the radiation dose is 100 mSv or higher, the cancer incidence probability is proportional to the radiation dose [1,2]. However, it is difficult to statistically evaluate the cancer incidence probability at doses of less than 100 mSv; that is, it is unclear how the cancer incidence probability increases or decreases with a radiation dose in this range [1,3,4,5,6,7,8,9,10]. The management of radiation risks is based on the hypotheses that radiation is dangerous to living organisms even at low doses and that the cancer incidence probability is proportional to a radiation dose below 100 mSv as well (linear non-threshold theory: LNT). However, there are doubts as to whether the LNT model is valid at low doses, and the phenomenon of radiation hormesis, in which radiation is beneficial to living organisms, has also been reported [11,12,13]. Therefore, it may be possible to clarify the truth at low doses by adding biological findings at the molecular–cellular level and mathematical models [11,14,15,16,17,18,19,20,21,22].

**Figure 1:**
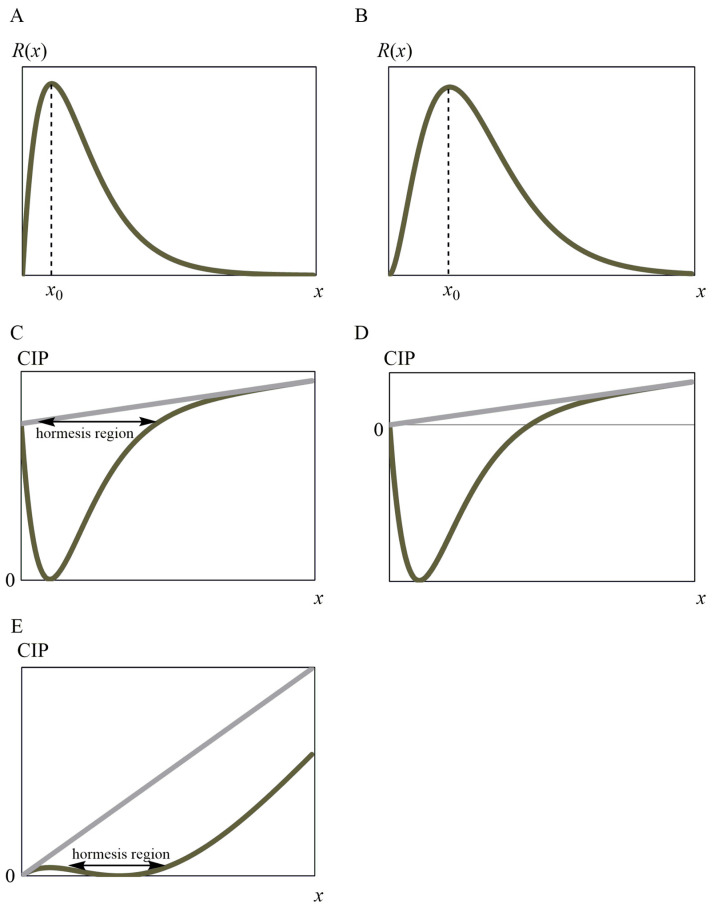
Graphs of *R*(*x*) and cancer incidence probability (CIP). These graphs were rewritten from the reference [31]. The radiation dose is defined as *x*. (**A**,**B**) The radiation dose at which *R*(*x*) reaches a maximum is defined as *x*_0_. (**A**) *R*(*x*) having a single point of inflection at *x* > *x*_0_. The graph of *R*(*x*) is hunchback-shaped. (**B**) *R*(*x*) having one point of inflection for 0 < *x* < *x*_0_ and another for *x* > *x*_0_. The graph of *R*(*x*) is mountain-shaped. (**C**–**E**) The black line indicates the graph of CIP considering *R*(*x*). The gray line indicates LNT. CIP = LNT − *R*(*x*). The two-way arrows indicate the hormesis region [31]. (**C**) The graph of CIP using *R*(*x*), which has a hunchback-shaped graph (**A**). CIP involves factors other than radiation (e.g., reactive oxygen species). (**D**) The virtual graph of CIP using *R*(*x*), which has a hunchback-shaped graph (**A**). CIP involves only radiation. If *R*(*x*) of (**A**) is used, CIP mathematically has negative values, and it is not real. (**E**) The graph of CIP using *R*(*x*), which has a mountain-shaped graph (**B**). CIP involves only radiation.

The radiation adaptive response has been reported as a phenomenon related to low dose [23,24,25,26,27,28,29,30], and the term “inhibition effect (*R*(*x*))” used in previous papers is not strictly equivalent to the adaptive response but is defined as the element that theoretically leads to radiation-specific hormesis-coupled LNT [31,32]. In addition, a hunchback-shaped graph has been proposed to describe components involved in the radiation adaptive response (Figure 1A) [33,34,35]. If such an increasing/decreasing component contributes to radiation hormesis, the cancer incidence probability must be greater than zero at 0 mSv (Figure 1C) [31,36,37,38,39]. In other words, the causes of cancer development in this case are not limited to radiation, and factors other than radiation [28,29] must be involved, for example, reactive oxygen species.

On the other hand, for cancers caused solely by radiation, the cancer incidence probability must be zero when the radiation dose is 0 mSv [31]. Moreover, if the components are generated solely by radiation, *R*(0) must be zero [31]. Therefore, *R*(*x*) with a mountain-shaped graph, as shown in Figure 1B, is required for the existence of a radiation hormesis effect (Figure 1E) [31]. If the *R*(*x*) of Figure 1A is used, the cancer incidence probability mathematically has negative values (Figure 1D), and it is impossible for the cancer incidence probability to have negative values. In Figure 1B, unlike Figure 1A, there is one inflection point in the region 0 < *x* < *x*_0_. In this paper, the mathematical equation used previously [31,32] is derived from the concept of chemical kinetics in Section 2, and a more general model is discussed in Section 3 and Section 4.

## 2. Generative Model Fitted to the Previously-Reported Equation

As one *R*(*x*) with a mountain-shaped graph (Figure 1B), Equation (1) has previously been used [31]. The maximum hormesis effect can be produced when *n* is 2 (Equation (2)) [31]. In fact, Fornalski [40,41,42,43,44] used Equations (1) and (2) as a function to show a radiation adaptive response. In this section, what model Equation (2) can be derived from is discussed.


(1) $$R\left(x\right)=x^{n}e^{-ax}$$

(2) $$R\left(x\right)=x^{2}e^{-ax}$$

**Scheme 1:**
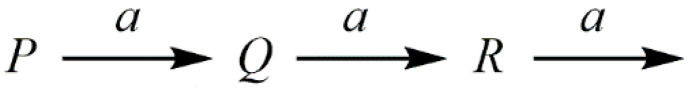
Sequential reaction when all rate constants are the same.

Usually, in reaction kinetics using differential equations, each component quantity is given as a function of time. Considering that the dose *x* is proportional to time if the dose rate is constant, each component quantity can be expressed as a function of dose *x*. Here, the reaction model of Scheme 1 is considered. In this scheme, the raw material *P* decomposes to produce *Q*, *Q* further decomposes to produce *R*, and *R* decomposes to another substance. In addition, when the component amount of each factor increases or decreases only with the dose of radiation, differential Equations (3)–(5) hold. All rate constants are the same, and the rate constant, *a*, is positive. Defining the initial quantity of *P*(*x*) as a positive number, *P*_0_, and solving these equations produces Equation (6). The obtained Equation (6) has the same form as Equation (2). This means that the component *R* produced in the second step of the sequential reaction is a candidate for the component that induces the hormesis effect.


(3) $$\frac{dP}{dx}=-aP$$

(4) $$\frac{dQ}{dx}=aP-aQ$$

(5) $$\frac{dR}{dx}=aQ-aR$$

(6) $$R\left(x\right)=\frac{1}{2}P_{0}a^{2}x^{2}e^{-ax}$$

## 3. Considerations Using a General Model

**Scheme 2:**
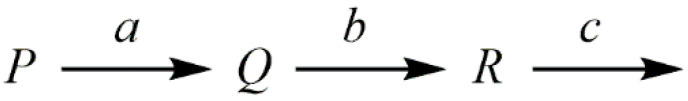
Sequential reaction with different rate constants.

In the previous section, it was shown that the component *R* in Scheme 1 obeys Equation (2). However, it is not usual that the three reaction rate constants are exactly the same. Therefore, we should consider a general model in which each reaction constant is different (Scheme 2). By solving the differential Equations (3), (7), and (8) in the same way as in Section 2, Equation (9) is obtained in accordance with a previous report [45]. Note that *P*_0_, *a*, *b*, and *c* are positive.


(7) $$\frac{dQ}{dx}=aP-bQ$$

(8) $$\frac{dR}{dx}=bQ-cR$$

(9) $$R\left(x\right)=P_{0}\frac{-ab}{\left(a-b\right)\left(b-c\right)\left(c-a\right)}\left\{\left(b-c\right)e^{-ax}+\left(c-a\right)e^{-bx}+\left(a-b\right)e^{-cx}\right\}$$

In Scheme 2, *Q*(*x*) can be easily derived mathematically to have a graph with the hunchbacked shape, as shown in Figure 1A; so, the component *Q* cannot induce the hormesis effect considered in this paper. On the other hand, if Equation (9) has a mountain-shaped graph (Figure 1B), as in Equation (2), then the component *R* is the candidate factor we desire. Therefore, in the next section, the shape of *R*(*x*) in Scheme 2 will be considered mathematically.

## 4. Shape of *R*(*x*) in Scheme 2: Proof That Equation (9) Has a Mountain-Shaped Graph as in Figure 1B

### 4.1. First-Order Differentiation

To prove that the graph of Equation (9) is mountain-shaped (Figure 1B), an increase/decrease in *R*(*x*) is considered. Consider function *f*(*x*) defined in Equation (10). Since *P*_0_, *a*, *b*, and *c* are positive, *abP*_0_ is always positive. For *R*(*x*)/*abP*_0_, *a*, *b*, and *c* can be treated symmetrically. Therefore, it is not necessary to consider all of *a* > *b* > *c*, *a* > *c* > *b*, *b* > *a* > *c*, *b* > *c* > *a*, *c* > *a* > *b*, and *c* > *b* > *a* but only one of these cases mathematically. That is, there is no loss of generality in considering *b* > *c* > *a* for *R*(*x*)/*abP*_0_. In this case, since −1/(*a* − *b*)(*b* − *c*)(*c* − *a*) is positive, we consider an increase/decrease in *f*(*x*).


(10) $$f\left(x\right)\;:=\left(b-c\right)e^{-ax}+\left(c-a\right)e^{-bx}+\left(a-b\right)e^{-cx}$$

Differentiation of *f*(*x*) leads to Equation (11).


(11) $$\frac{df}{dx}=e^{-ax}\left\{a\left(c-b\right)+b\left(a-c\right)e^{\left(a-b\right)x}+c\left(b-a\right)e^{\left(a-c\right)x}\right\}$$

Next, we consider the function *g*(*x*) defined in Equation (12); note that the sign of *g*(*x*) for a given *x* is the same as that of *df*/*dx*.


(12) $$g\left(x\right)\;:=a\left(c-b\right)+b\left(a-c\right)e^{\left(a-b\right)x}+c\left(b-a\right)e^{\left(a-c\right)x}$$

Differentiation of *g*(*x*) gives Equation (13).


(13) $$\frac{dg}{dx}=\left(c-a\right)\left(b-a\right)e^{ax}\left(be^{-bx}-ce^{-cx}\right)$$

Since we have assumed *b* > *c* > *a*, Equation (14) must hold for *dg*/*dx* to be nonnegative.


(14) $$be^{-bx}-ce^{-cx}\geq 0$$

Solving Equation (14) yields Equation (15), which also defines *x*_1_.


(15) $$x\leq \frac{\ln\frac{b}{c}}{b-c}=:\;x_{1}$$

From Equation (12), the following values are obtained when *x* is 0 and when *x* is infinite.


(16) $$g\left(0\right)=0$$

(17) $$g\left(\infty \right)=a\left(c-b\right)<0$$

Summarizing the above, a derivative sign chart of *g*(*x*) is presented in Table 1.

From the signs in Table 1, there is only one value of *x* for which *g*(*x*) is zero. If such a value of *x* is defined as *x*_2_, Equation (18) holds. This *x*_2_ is larger than *x*_1_, and thus the signs of *df*/*dx* are derived from the signs of *g*(*x*), which in turn give us the signs of *dR*/*dx* (Table 2).


(18) $$g\left(x_{2}\right)=0$$

### 4.2. Second-Order Differentiation

Further differentiation of Equation (11) yields Equation (19).


(19) $$\frac{d^{2}f}{dx^{2}}=e^{-ax}\left\{a^{2}\left(b-c\right)+b^{2}\left(c-a\right)e^{\left(a-b\right)x}+c^{2}\left(a-b\right)e^{\left(a-c\right)x}\right\}$$

Considering the function *h*(*x*) defined in Equation (20), we see that the sign of *h*(*x*) at a given *x* is the same as that of *d*^2^*f*/*dx*^2^.


(20) $$h\left(x\right)\;:=a^{2}\left(b-c\right)+b^{2}\left(c-a\right)e^{\left(a-b\right)x}+c^{2}\left(a-b\right)e^{\left(a-c\right)x}$$

Differentiation of *h*(*x*) produces Equation (21).


(21) $$\frac{dh}{dx}=\left(c-a\right)\left(b-a\right)e^{ax}\left(c^{2}e^{-cx}-b^{2}e^{-bx}\right)$$

Since we have assumed *b* > *c* > *a*, Equation (22) must hold for *dh*/*dx* to be nonnegative.


(22) $$c^{2}e^{-cx}-b^{2}e^{-bx}\geq 0$$

Solving Equation (22) yields Equation (23). Using the definition in Equation (15), we can also express the relation in Equation (15) using *x*_1_.


(23) $$x\geq 2\frac{\ln\frac{b}{c}}{b-c}=2x_{1}$$

From Equation (20), the following values are obtained when *x* is 0 and when *x* is infinite.


(24) $$h\left(0\right)=\left(b-c\right)\left(b-a\right)\left(c-a\right)>0$$

(25) $$h\left(\infty \right)=a^{2}\left(b-c\right)>0$$

A derivative sign chart of *h*(*x*) summarizing the above is presented in Table 3.

It remains necessary to find the sign of *h*(2*x*_1_). Substituting 2*x*_1_ for *x* in Equation (20), we obtain Equation (26).


(26) $$h\left(2x_{1}\right)=a^{2}\left(b-c\right)+b^{2}\left(c-a\right)e^{2\left(a-b\right)x_{1}}+c^{2}\left(a-b\right)e^{2\left(a-c\right)x_{1}}$$

Equation (27) is obvious from Equations (22) and (23). Substituting Equation (27) into Equation (26) and rearranging, we obtain Equation (28).


(27) $$c^{2}e^{-2cx_{1}}-b^{2}e^{-2bx_{1}}=0$$

(28) $$h\left(2x_{1}\right)=\left(b-c\right)\left(a+be^{\left(a-b\right)x_{1}}\right)\left(a-be^{\left(a-b\right)x_{1}}\right)$$

Once the sign of *m*(*x*_1_), defined by Equation (29), is known, the sign of *h*(2*x*_1_) is clear.


(29) $$m\left(x_{1}\right)\;:=a-be^{\left(a-b\right)x_{1}}$$

Applying Equation (15) to Equation (29), we can obtain Equation (30).


(30) $$m\left(x_{1}\right)\;:=a-b^{\frac{a-c}{b-c}}c^{\frac{b-a}{b-c}}$$

Next, we consider the function *p*(*k*) defined by Equation (31). Note that *p*(*k*) decreases monotonically with *k* for *k* > 0.


(31) $$p\left(k\right)=\frac{\ln k}{k-1}\;(k>0)$$

We recall that *a* is positive. The assumption *b* > *c* > *a* implies that *b*/*a* > *c*/*a*; so, the monotonicity of Equation (31) implies that Equation (32) holds.


(32) $$\frac{\ln\frac{b}{a}}{\frac{b}{a}-1}<\frac{\ln\frac{c}{a}}{\frac{c}{a}-1}$$

Rearranging Equation (32), we can obtain Equation (33); so, *m*(*x*_1_) is negative.


(33) $$a-b^{\frac{a-c}{b-c}}c^{\frac{b-a}{b-c}}<0$$

Hence, *h*(2*x*_1_) is negative, and we can update Table 3 as Table 4.

From the signs in Table 4, there are two values of *x* for which *h*(*x*) is zero. The *x* value in the range 0 < *x* < 2*x*_1_ is defined as *x*_3_ and that in the range 2*x*_1_ < *x* is defined as *x*_4_, expressed as Equations (34) and (35), respectively. We then derive the signs of *d*^2^*f*/*dx*^2^ from those of *h*(*x*), which are the same as those of *d*^2^*R*/*dx*^2^ (Table 5).

(34) $$h\left(x_{3}\right)=0$$

(35) $$h\left(x_{4}\right)=0$$

### 4.3. Increase/Decrease in R(x)

Using Table 2 and Table 5, we now consider the increase/decrease in *f*(*x*). Recall that *x*_3_ < *x*_4_. If *d*^2^*f*/*dx*^2^(*x*_2_) > 0, then either (i) or (iii) below is correct, whereas if *d*^2^*f*/*dx*^2^(*x*_2_) < 0, then (ii) is correct.

(i) *x*_2_ < *x*_3_ < *x*_4_;
(ii) *x*_3_ < *x*_2_ < *x*_4_;
(iii) *x*_3_ < *x*_4_ < *x*_2_.

Substituting *x*_2_ for *x*, we obtain Equation (36) from Equation (11) and Equation (37) from Equation (19). For convenience, we define *d*^2^*f*/*dx*^2^(*x*_2_) as *u*. We want to find the sign of *u*.


(36) $$\frac{df}{dx}\left(x_{2}\right)=e^{-ax_{2}}\left\{a\left(c-b\right)+b\left(a-c\right)e^{\left(a-b\right)x_{2}}+c\left(b-a\right)e^{\left(a-c\right)x_{2}}\right\}=0$$

(37) $$\frac{d^{2}f}{dx^{2}}\left(x_{2}\right)=e^{-ax_{2}}\left\{a^{2}\left(b-c\right)+b^{2}\left(c-a\right)e^{\left(a-b\right)x_{2}}+c^{2}\left(a-b\right)e^{\left(a-c\right)x_{2}}\right\}=:u$$

Substituting Equation (36) into Equation (37) gives Equation (38).


(38) $$u=\left(c-a\right)\left(b-a\right)\left(be^{-bx_{2}}-ce^{-cx_{2}}\right)$$

Next, we define *v* as in Equation (39). From the assumption *b* > *c* > *a*, the sign of *v* tells us the sign of *u*.


(39) $$v\;:=be^{-bx_{2}}-ce^{-cx_{2}}$$

From Equations (14) and (15), we can obtain Equation (40).


(40) $$be^{-bx_{1}}=ce^{-cx_{1}}$$

We multiply both sides of Equation (39) by *x*_2_ and both sides of Equation (40) by *x*_1_.


(41) $$x_{2}v=bx_{2}e^{-bx_{2}}-cx_{2}e^{-cx_{2}}$$

(42) $$bx_{1}e^{-bx_{1}}=cx_{1}e^{-cx_{1}}$$

Note that *x*_1_ < *x*_2_ and *c* < *b*; so, either (iv) or (v) below is correct.

(iv) *cx*_1_ < *cx*_2_ < *bx*_1_ < *bx*_2_;
(v) *cx*_1_ < *bx*_1_ < *cx*_2_ < *bx*_2_.

Now, we define *q*(*k*) as in Equation (43) and define *k*_1_ as the maximizer of *q*(*k*).


(43) $$q\left(k\right)=ke^{-k}\;\left(k\geq 0\right)$$

**Figure 2:**
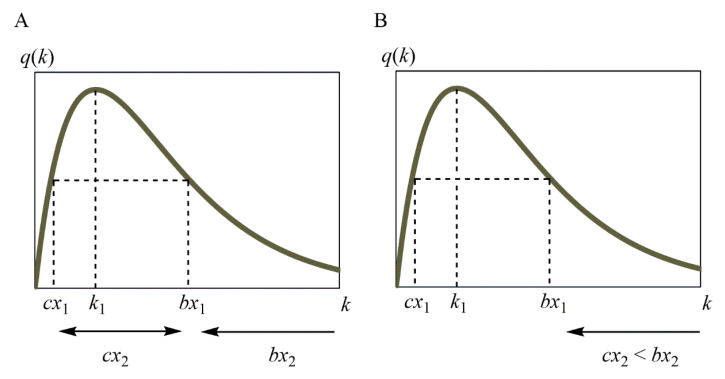
Graphs of *q*(*k*). *q*(*cx*_1_) is equal to *q*(*bx*_1_). The maximizer of *q*(*k*) is defined as *k*_1_. (**A**) (iv) *cx*_1_ < *cx*_2_ < *bx*_1_ < *bx*_2_. *q*(*cx*_2_) is greater than *q*(*bx*_1_) and *q*(*cx*_1_). *q*(*bx*_2_) is less than *q*(*bx*_1_). (**B**) (v) *cx*_1_ < *bx*_1_ < *cx*_2_ < *bx*_2_. *q*(*cx*_2_) is less than *q*(*bx*_1_) and *q*(*cx*_1_). *q*(*bx*_2_) is less than *q*(*cx*_2_).

Considering Equation (42) and the increase/decrease in *q*(*k*), *cx*_1_ is smaller and *bx*_1_ is larger than *k*_1_ in the graph shown in Figure 2. We now compare *q*(*cx*_1_), *q*(*bx*_1_), *q*(*cx*_2_), and *q*(*bx*_2_) for the cases in (iv) and (v).

Given *cx*_1_ < *cx*_2_ < *bx*_1_ from (iv), we obtain *q*(*cx*_2_) > *q*(*bx*_1_) = *q*(*cx*_1_). Moreover, *q*(*bx*_1_) > *q*(*bx*_2_) is established from *bx*_1_ < *bx*_2_. Therefore, *q*(*cx*_2_) > *q*(*bx*_2_) holds.

If instead we assume *cx*_1_ < *bx*_1_ < *cx*_2_ < *bx*_2_ as in (v), we obtain *q*(*cx*_1_) = *q*(*bx*_1_) > *q*(*cx*_2_) > *q*(*bx*_2_). Therefore, we have *q*(*cx*_2_) > *q*(*bx*_2_).

Thus, Equation (44) holds in both cases (iv) and (v).


(44) $$cx_{2}e^{-cx_{2}}>bx_{2}e^{-bx_{2}}$$

From Equation (44), *v* is negative, and consequently, *u* is negative. Therefore, (ii) is correct. From the order *x*_3_ < *x*_2_ < *x*_4_ and Table 2 and Table 5, the derivative sign chart of *R*(*x*) is as shown in Table 6. It is thus proved that Equation (9) is mountain-shaped, as shown in Figure 1B.

## 5. Conclusions and Implications

According to the proof described in Section 4, Equation (9) has a mountain-shaped graph with two inflection points. Therefore, Equation (9) can be used instead of Equation (2) for the radiation-specific component *R* that can induce the hormesis effect. In other words, in the case of cancer caused solely by radiation and in a general sequential reaction (Scheme 2) with different rate constants for each step, the component *R* that is produced in the second step may induce the radiation-specific hormesis effect at low doses. What we find in this paper is that at the present time there is no biophysical background other than Scheme 1 and Scheme 2 and only a theoretical mathematical approach for understanding Equations (2), (6), and (9). Therefore, this approach on some real data [46] should be used and tested. Furthermore, the exploration and overexpression of such a component may lead to the development of cancer-reducing therapeutic modalities.

## Figures and Tables

**Table 1:** The derivative sign chart of *g*(*x*).

x	0	⋯	$x_{1}$	⋯	∞
$\frac{dg}{dx}$		+	0	−	
$g\left(x\right)$	0	up		down	−

**Table 2:** The signs of *df*/*dx* and *dR*/*dx*.

x	0	⋯	$x_{2}$	⋯	∞
$\frac{df}{dx}$	0	+	0	−	
$\frac{dR}{dx}$	0	+	0	−	

**Table 3:** The derivative sign chart of *h*(*x*) without *h*(2*x*_1_).

x	0	⋯	$2x_{1}$	⋯	∞
$\frac{dh}{dx}$		−	0	+	
$h\left(x\right)$	+	down	$\left(unknown\right)$	up	+

**Table 4:** The full derivative sign chart of *h*(*x*).

x	0	⋯	$2x_{1}$	⋯	∞
$\frac{dh}{dx}$		−	0	+	
$h\left(x\right)$	+	down	−	up	+

**Table 5:** The signs of *d*^2^*f*/*dx*^2^ and *d*^2^*R*/*dx*^2^.

x	0	⋯	$x_{3}$	⋯	$x_{4}$	⋯
$\frac{d^{2}f}{dx^{2}}$		+	0	−	0	+
$\frac{d^{2}R}{dx^{2}}$		+	0	−	0	+

**Table 6:** The derivative sign chart of *R*(*x*).

x	0	⋯	$x_{3}$	⋯	$x_{2}$	⋯	$x_{4}$	⋯
$\frac{dR}{dx}$		+		+	0	−		−
$\frac{d^{2}R}{dx^{2}}$		+	0	−		−	0	+
$R\left(x\right)$	0	up, v		up, ^		down, ^		down, v

Note of Table 6: “v” means concave upward. “^” means concave downward.

## Data Availability

Not applicable.

## References

[1] ICRP (2006). Low-Dose Extrapolation of Radiation-Related Cancer Risk. ICRP Publication 99. International Commission on Radiological Protection.

[2] National Research Council (2005). Health Risks from Exposure to Low Levels of Ionizing, Radiation. BEIR VII-Phase 2.

[3] UNSCEAR (2010). Summary of Low-Dose Radiation Effects on Health. UNSCEAR 2010 Report.

[4] Tubiana M., Arengo A., Averbeck D., Masse R. (2007). Low-dose risk assessment. Radiat. Res..

[5] Mossman K.L. (2008). Economic and policy considerations drive the LNT debate. Radiat. Res..

[6] Leonard B.E. (2008). Common sense about the linear no-threshold controversy-give the general public a break. Radiat. Res..

[7] Tubiana M., Aurengo A., Averbeck D., Masse R. (2008). Low-dose risk assessment: The debate continues. Radiat. Res..

[8] Feinendegen L.E., Paretzke H., Neumann R.D. (2008). Two principal considerations are needed after low doses of ionizing radiation. Radiat. Res..

[9] Mothersill C., Seymour C. (2022). Low dose radiation mechanisms: The certainty of uncertainty. Mutat. Res. Genet. Toxicol. Environ. Mutagen..

[10] Boice J.D.J. (2017). The linear nonthreshold (LNT) model as used in radiation protection: An NCRP update. Int. J. Radiat. Biol..

[11] Socol Y., Dobrzynski L., Doss M., Feinendegen L.E., Janiak M.K., Miller M.L., Sanders C.L., Scott B.R., Ulsh B., Vaiserman A. (2014). Commentary: Ethical issues of current health-protection policies on low-dose ionizing radiation. Dose Response.

[12] Luckey T.D. (1991). Radiation Hormesis.

[13] Sanders C.L. (2010). Radiation Hormesis and the Linear-No-Threshold Assumption.

[14] UNSCEAR (2000). Sources and Effects of Ionizing Radiation. United Nations Scientific Committee on the Effects of Atomic Radiation. UNSCEAR 2000 Report.

[15] NCRP (2001). Evaluation of the Linear-Nonthreshold Dose-Response Model for Ionizing Radiation.

[16] Uchinomiya K., Yoshida K., Kondo M., Tomita M., Iwasaki T. (2020). A mathematical model for stem cell competition to maintain a cell pool injured by radiation. Radiat. Res..

[17] Kim S.B., Sanders N. (2017). Model averaging with AIC weights for hypothesis testing of hormesis at low doses. Dose Response.

[18] Kim S.B., Bartell S.M., Gillen D.L. (2016). Inference for the existence of hormetic dose-response relationships in toxicology studies. Biostatistics.

[19] Esposito G., Campa A., Pinto M., Simone G., Tabocchini M.A., Belli M. (2011). Adaptive response: Modelling and experimental studies. Radiat. Prot. Dosim..

[20] Wodarz D., Sorace R., Komarova N.L. (2014). Dynamics of cellular responses to radiation. PLoS Comput. Biol..

[21] Socol Y., Shaki Y.Y., Dobrzyński L. (2020). Damped-oscillator model of adaptive response and its consequences. Int. J. Low Rad..

[22] Smirnova O.A., Yonezawa M. (2003). Radioprotection effect of low level preirradiation on mammals: Modeling and experimental investigations. Health Phys..

[23] Olivieri G., Bodycote J., Wolff S. (1984). Adaptive response of human lymphocytes to low concentrations of radioactive thymidine. Science.

[24] Wolff S. (1998). The adaptive response in radiobiology: Evolving insights and implications. Environ. Health Perspect..

[25] Azzam E.I., Raaphorst G.P., Mitchel R.E. (1994). Radiation-induced adaptive response for protection against micronucleus formation and neoplastic transformation in C3H 10T1/2 mouse embryo cells. Radiat. Res..

[26] Shadley J.D. (1994). Chromosomal adaptive response in human lymphocytes. Radiat. Res..

[27] Thierens H., Vral A., Barbé M., Meijlaers M., Baeyens A., Ridder L.D. (2002). Chromosomal radiosensitivity study of temporary nuclear workers and the support of the adaptive response induced by occupational exposure. Int. J. Radiat. Biol..

[28] Tapio S., Jacob V. (2007). Radioadaptive response revisited. Radiat. Environ. Biophys..

[29] Guéguen Y., Bontemps A., Ebrahimian T.G. (2019). Adaptive responses to low doses of radiation or chemicals: Their cellular and molecular mechanisms. Cell Mol. Life Sci..

[30] Devic C., Ferlazzo M.L., Foray N. (2018). Influence of individual radiosensitivity on the adaptive response phenomenon: Toward a mechanistic explanation based on the nucleo-shuttling of atm protein. Dose-Response.

[31] Kino K. (2020). The prospective mathematical idea satisfying both radiation hormesis under low radiation doses and linear non-threshold theory under high radiation doses. Gene Environ..

[32] Kino K., Ohshima T., Takeuchi H., Kobayashi T., Kawada T., Morikawa M., Miyazawa H. (2021). Considering existing factors that may cause radiation hormesis at <100 mSv and obey the linear no-threshold theory at ≥100 mSv. Challenges.

[33] Feinendegen L.E. (2005). Evidence for beneficial low level radiation effects and radiation hormesis. Br. J. Radiol..

[34] Feinendegen L.E. (2005). Low doses of ionizing radiation: Relationship between biological benefit and damage induction. A synopsis. World J. Nucl. Med..

[35] Agathokleous E., Kitao M., Calabrese E.J. (2018). Environmental hormesis and its fundamental biological basis: Rewriting the history of toxicology. Environ. Res..

[36] Calabrese E.J., Shamoun D.Y., Hanekamp J.C. (2015). Cancer risk assessment: Optimizing human health through linear dose-response models. Food Chem. Toxicol..

[37] Lampe N., Breton V., Sarramia D., Sime-Ngando T., Biron D.G. (2017). Understanding low radiation background biology through controlled evolution experiments. Evol. Appl..

[38] Sutou S. (2016). A message to Fukushima: Nothing to fear but fear itself. Genes Environ..

[39] Scott B.R. (2011). Residential radon appears to prevent lung cancer. Dose Response.

[40] Fornalski K.W. (2014). Mechanistic model of the cells irradiation using the stochastic biophysical input. Int. J. Low Radiat..

[41] Dobrzyński L., Fornalski K.W., Reszczyńska J., Janiak M.K. (2019). Modeling cell reactions to ionizing radiation: From a lesion to a cancer. Dose Response.

[42] Fornalski K.W., Adamowski Ł., Dobrzyński L., Jarmakiewicz R., Powojska A., Reszczyńska J. (2022). The radiation adaptive response and priming dose influence: The quantification of the Raper-Yonezawa effect and its three-parameter model for postradiation DNA lesions and mutations. Radiat. Environ. Biophys..

[43] Dobrzyński L., Fornalski K.W., Socol Y., Reszczyńska J.M. (2016). Modeling of irradiated cell transformation: Dose-and time-dependent effects. Radiat. Res..

[44] Fornalski K.W. (2019). Radiation adaptive response and cancer: From the statistical physics point of view. Phys. Rev. E.

[45] Bateman H. (1910). Solution of a system of differential equations occurring in the theory of radio-active transformations. Proc. Cambridge Phil. Soc..

[46] UNSCEAR (1994). Sources and Effects of Ionizing Radiation. UNSCEAR 1994 Report.

